# Light scattering measured with spatial frequency domain imaging can predict stromal versus epithelial proportions in surgically resected breast tissue

**DOI:** 10.1117/1.JBO.24.7.071605

**Published:** 2018-09-27

**Authors:** David M. McClatchy, Elizabeth J. Rizzo, Wendy A. Wells, Candice C. Black, Keith D. Paulsen, Stephen C. Kanick, Brian W. Pogue

**Affiliations:** a Dartmouth College, Thayer School of Engineering, Hanover, New Hampshire, United States; b Dartmouth College, Geisel School of Medicine, Department of Pathology, Hanover, New Hampshire, United States; c Norris Cotton Cancer Center, Dartmouth-Hitchcock Medical Center, Lebanon, New Hampshire, United States

**Keywords:** breast conserving surgery, spatial frequency domain imaging, light scattering, optical properties, digitized histology

## Abstract

This study aims to determine if light scatter parameters measured with spatial frequency domain imaging (SFDI) can accurately predict stromal, epithelial, and adipose fractions in freshly resected, unstained human breast specimens. An explicit model was developed to predict stromal, epithelial, and adipose fractions as a function of light scattering parameters, which was validated against a quantitative analysis of digitized histology slides for N=31 specimens using leave-one-out cross-fold validation. Specimen mean stromal, epithelial, and adipose volume fractions predicted from light scattering parameters strongly correlated with those calculated from digitized histology slides (r=0.90, 0.77, and 0.91, respectively, p-value $<1\times 10^{-6}$). Additionally, the ratio of predicted epithelium to stroma classified malignant specimens with a sensitivity and specificity of 90% and 81%, respectively, and also classified all pixels in malignant lesions with 63% and 79%, at a threshold of 1. All specimens and pixels were classified as malignant, benign, or fat with 84% and 75% accuracy, respectively. These findings demonstrate how light scattering parameters acquired with SFDI can be used to accurately predict and spatially map stromal, epithelial, and adipose proportions in fresh unstained, human breast tissue, and suggest that these estimations could provide diagnostic value.

## 1 Introduction

Breast conserving surgery (BCS) combined with radiation therapy is becoming an increasingly popular treatment for localized breast cancer. However, it still remains a challenge for surgeons to obtain clear surgical margins, with 20% to 40% of patients requiring follow-up re-excision procedures.^1^ While histopathology is the “gold-standard” for determining the margin status, it can take multiple days to process and analyze a specimen, which requires specimen sectioning, dehydrating, fixing, paraffin embedding, microtoming, slide mounting, and slide staining. Because of this challenge, there has been a great effort to determine novels ways to rapidly triage breast tissue specimens, with the ultimate goal of intraoperative tumor margin assessment.

A common technique to overcome this time barrier is frozen section pathology (FSP), where cut tissue specimens are flash frozen and mounted during the surgery, to have slides diagnosed by a pathologist before closing the surgical cavity. While this technique has shown some diagnostic value despite known imaging artifacts from freezing,^2^ it remains time consuming (20 to 25 min for a single round of FSP,^3^ 50 minutes for eight FSP sections^4^), costly, and stills suffers from under sampling of the tissue. There have been promising advances in various novel microscopy techniques, which can create virtual histology slides in thick unprocessed tissue, with^5^^,^^6^ or without^7^^,^^8^ the application of topical fluorescent dyes. And similarly, optical coherence tomography can provide microscopic resolution in unprocessed human breast tissue.^9^^,^^10^ However, in the BCS workflow, there is still an issue of under sampling with these techniques, as the time it takes to scan and evaluate microscopic fields of view over an entire lumpectomy specimen creates a clinical translation challenge.

Wide-field optical imaging can provide rapid sensing of an entire specimen surface, and while it lacks microscopic resolution, it can provide molecular or morphological sensitivity.^11^^,^^12^ In particular, label-free reflectance imaging techniques utilizing multispectral and/or structured illumination have employed machine learning techniques to back out tissue diagnoses from raw reflectance spectra or optical properties.^13^^,^^14^ Although machine learning can act as a conduit between raw optical signals and tissue diagnoses, the cloaking of the biological mechanism between the source data and predicted pathology can often hinder clinical acceptance of these technologies. And although optical properties related to light scattering have specifically shown enhanced sensitivity to tissue morphology,^14^^–^^16^ these parameters lack a clear clinical or biological definition.

Therefore, the ultimate purpose of this study is to elucidate the relationship between clinically relevant histological features from optical reflectance signatures, and furthermore develop and validate a method to predict these histological features from optical signals. For this study, a cohort of N=31 freshly resected human breast tissue specimens was imaged with a spatial frequency domain imaging (SFDI) system, resulting in light scattering parameter maps coregistered to histopathology. The explicit relationships between optical scattering properties and stromal, epithelial, and adipose volume fractions segmented from coregistered digitized histopathology are presented. Furthermore, a model by which these histologic features can be predicted is validated and its diagnostic potential in distinguishing malignant from normal human breast tissue is demonstrated.

## 2 Materials and Methods

### 2.1 Imaging of Fresh Breast Tissue Samples Study Protocol

An *ex vivo* breast specimen imaging study conducted in the Department of Pathology at Dartmouth Hitchcock Medical Center (DHMC) was approved by the Institutional Review Board for the protection of human subjects as detailed in a previous publication.^15^ To briefly summarize, excised breast tissue from patients undergoing elected and consented breast surgery was immediately transported to the Department of Pathology. Tissue that was in excess of what was needed to make a clinical diagnosis and designated for the tissue-bank was considered for imaging in this study. Tissues that were grossly identified as invasive cancer, fibroglandular, fibroadenoma, or adipose were cut to a size of roughly 25  mm×25  mm×5  mm, placed on a glass slide, and immediately imaged from below with a commercial SFDI system (Modulated Imaging, Inc.),^17^^,^^18^ described in Sec. 2.2. After imaging, the specimens were immediately returned to the Department of Pathology and underwent standard histological processing of dehydration, fixation, paraffin embedding, slide mounting, and staining with hematoxylin and eosin (H&E). The resulting H&E slides were read by an expert pathologist (W.A.W.) and included in the patient’s report.

In total, 37 specimens were imaged, which represents an expanded dataset obtained from a previous study,^15^ where only 22 specimens were collected. Furthermore, fibroadenoma (N=5) and mucinous ductal carcinoma (N=1) specimens were excluded from this study. Theses diagnoses have unique histopathologic features that were not amenable to the color-based stroma and epithelium segmentation analysis described in Sec. 2.3. Thus, N=10 invasive cancer specimens were included [N=7 invasive ductal (IDc), N=2 intralobular (ILc), and N=1 male invasive ductal IDc], N=14 normal fibroglandular specimens [N=2 with fibrocystic disease (FCD) and N=1 male gynecomastia], and N=5 pure fat specimens. A total of N=31 specimens were included as shown in Table 1.

**Table 1:** A table listing patient and specimen sample sizes.

Class	Subtype	$N_{\text{patients}}$	$N_{\text{specimens}}$	$n_{\text{pixels}}$
Invasive cancer		10	10	12,225
	IDc	7	7	7841
	ILc	2	2	4203
	Male IDc	1	1	181
Fibroglandular		14	16	29,233
	Normal breast	11	13	24,373
	Normal breast w. FCD	2	2	1319
	Gynecomastia	1	1	1762
Fat		5	5	9073
Total		29	31	50,521

### 2.2 Spatial Frequency Domain Imaging and Light-Scattering Parameters

The SFDI system and light-scattering parameters are thoroughly described in previous publications.^15^^,^^19^ A description of the main features pertaining to this study is summarized below. The SFDI system utilized three light-emitting diodes at λ = [658, 730, and 850] nm, which focused light on a digital micromirror device (DMD) to sequentially project sinusoidal intensity patterns with spatial frequencies, $f_{x}$, in the range of [0 to 0.2 and 0.5 to 0.9] ${\mathrm{mm}}^{-1}$ and in increments of $0.05\;{\mathrm{mm}}^{-1}$ onto the tissue surface. A charged coupled device (CCD) camera captured the remitted intensity patterns. Offline, the image stacks were read into MATLAB (v2016a, Mathworks, Inc.) and demodulated and calibrated, which yielded reflectance maps over the acquired wavelengths and spatial frequencies. These reflectance maps were inverted into maps of optical properties through a pixel-by-pixel least square fitting routine, *lsqnonlin* (MATLAB, v2016a), which minimized the difference between measured reflectance and light propagation model predicted reflectance. For the forward light propagation models, diffusion theory in the spatial frequency domain^18^ is used for $f_{x}<0.2\;{\mathrm{mm}}^{-1}$ and a semiempirical subdiffusive model^19^ is used for $f_{x}>0.5\;{\mathrm{mm}}^{-1}$.

The resulting optical properties of interest quantifying light scattering were the phase function parameter γ, reduced scattering coefficient $\mu_{s}^{\prime }$, and wavelength versus scatter power, B. The absorption coefficient, $\mu_{a}$, was also quantified but was not included in this analysis as chromophore mapping in BCS specimens was the subject of previous studies, which demonstrated that spectroscopic scattering parameters were more diagnostically discriminant.^14^ The phase function parameter quantified directional light scattering (relative amount of forward to backward scatter) and is related to the amount of scattering features smaller and larger than the wavelength of light.^20^^,^^21^ The reduced scattering coefficient is related to the overall density of scattering features.^22^ The scatter power quantifies the exponential power of which $\mu_{s}^{\prime }$ varies with the wavelength of light {assuming $\mu_{s}^{\prime }(\lambda )=A{\left[\lambda /(800\;\mathrm{nm})\right]}^{-B}$}, and has been shown to be related to size-scale distribution of scattering features.^23^^,^^24^ Previous studies have shown the spatial resolution of $\mu_{s}^{\prime }$ to be ∼2  mm with a 1- to 2-mm depth penetration, while both the spatial resolution and depth penetration and γ was <1  mm.^15^

### 2.3 Quantitative Digital Histology Analysis

**Fig. 1:**
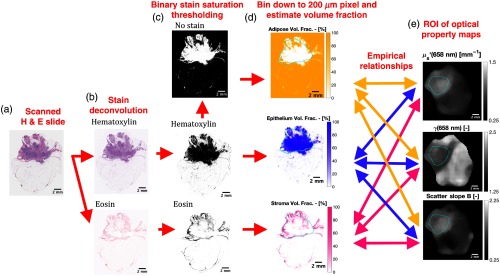
(a) A digitally scanned H&E slide with resolution of 500 pixels/mm. (b) Intensity images of the hematoxylin and eosin stains calculated through a color deconvolution of the H&E slide. (c) Binary hematoxylin, eosin, and no stain images calculated from a manual threshold of the color saturation of the deconvolded stain intensity images (note: black = foreground, white = background). (d) Volume fractions estimates of epithelium, stroma, and adipose calculated over 200-μm×200-μm areas of the binary stain images. (e) Corresponding optical property maps of the fresh specimen acquired prefixation and prestaining. The lesion outlined in cyan in both (d) and (e) denotes the region over which empirical relationships are determined between the histological and optical data.

The goal of the color-based quantitative histological analysis was to estimate the relative fraction of epithelium, stroma, and adipose over each lesion, assuming that these tissue specimens were comprised of these three bulk tissue morphologies. Physical H&E stained histology slides of the study specimens were digitized with a slide scanner with a resolution of 500 or 1000 pixels per mm. The methodology of processing the digitized H&E slides is shown in Figs. 1(a)–1(d). First, scanned slides underwent white balancing to control for minor changes in imaging conditions. Next, slides were deconvolved into monochromatic hematoxylin and eosin intensity channels as shown in Fig. 1(b), using a well-established spectral unmixing technique.^25^ This was implemented using the “Color Deconvolution” plugin (v1.7) in Fiji (v2.0.0): a distribution of the open-source image processing software package ImageJ.^26^ Next, the hematoxylin and eosin intensity images were converted to binary images, shown in Fig. 1(c), from which volume fractions of epithelium, stroma, and adipose were estimated. The binary conversion process was as follows: hematoxylin and eosin intensity images were converted from a RGB (red, green, and blue) to HSV (hue, saturation, and value) color space using the *rgb2hsv* function in MATLAB (v2016a), and the color saturation was used to compare each stain’s affinity. Pixels with greater hematoxylin saturation than eosin saturation were labeled hematoxylin, whereas pixels with greater eosin than hematoxylin saturation where labeled eosin. Pixels without significant hematoxylin or eosin saturation (<1/10th maximum value) being white space were labeled as no stain. Next, epithelium, stroma, and adipose volume fractions, shown in Fig. 1(d), were estimated from these binary stain images as the fraction of hematoxylin, eosin, or no stain labeled pixels, respectively, within 200-μm×200-μm voxels. Because of distortions through histological processing steps (e.g., dehydration and fixation), exact pixel-to-pixel correlation between the histological and optical maps could not be made. Instead, a certified and expert histolopathologist (W.A.W.) outlined a single lesion on each digitized histology slide, which was used to qualitatively inform a conservative ROI selected within the lesion of the histological fraction maps and optical property maps, as outlined in cyan in Figs. 1(d) and 1(e). The mean and standard deviation were calculated for each optical property and histological ROI and used for the model fitting, with each data point corresponding to a specific specimen. The ROI in the histology map are slightly different from the ROI in the optical property due to the changes in shape induced by the fixation process, but the authors note that the minor deviations in the shape of the ROI were nearly insensitive to the results as the mean of the ROI was analyzed rather a pixel-by-pixel analysis.

### 2.4 Optical-Histological Model Creation

**Fig. 2:**
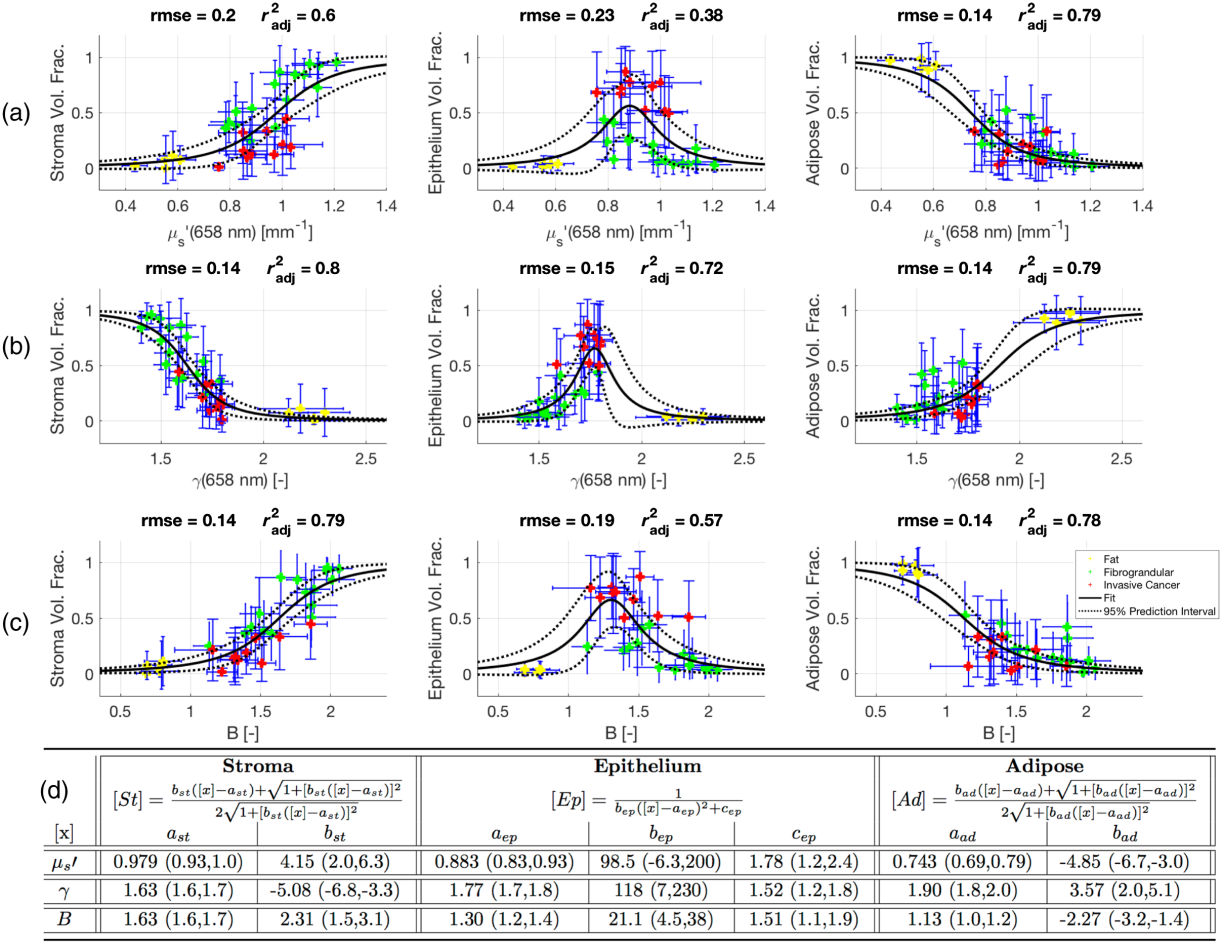
A summary of the relationships between the volume fractions of stroma, epithelium, and adipose calculated from the H&E sections and the optical properties (a) $\mu_{s}^{\prime }$, (b) γ, and (c) B, which describe overall light scattering intensity, intensity change with scatter angle, and intensity change with wavelength of light, respectively. The rmse and the adjusted coefficient of determination ($r_{\mathrm{adj}}^{2}$) are shown for each relationship. The solid line is the fitted equation and the dotted line is 95% prediction interval. Data points represent means within each specimen, while error-bars represent one standard deviation. In (d), the equations for fitting the volume fractions of stroma, epithelium, and adipose as a function of each optical property, denoted by [x], are shown, along with the values of the fitted parameters. Values in parenthesis are 95% (CIs).

With the paired light scattering parameters and segmented epithelium, stroma, and adipose fractions, models were created using the fit function in the Curve Fitting Toolbox in MATLAB (v2016a). The mean epithelium, stroma, and adipose fractions are plotted as functions of $\mu_{s}^{\prime }$ in Fig. 2(a), γ in Fig. 2(b), and B in Fig. 2(c), respectively, where each data point represents each specimen mean and the error-bars represent one standard deviation. The fitted relationships are shown as solid lines and the 95% prediction intervals are shown as dotted lines. The explicit form of each relationship is shown in Fig. 2(d), along with the specific fitted parameters and corresponding 95% confidence intervals (CIs) of each parameter. Given the monotonic nature of the stroma and adipose volume fractions, a two-parameter logistic-like equation well described the data. A three-parameter Gaussian-like equation described the peaked epithelium volume fraction response, with fat and fibroglandular specimens both having a diminished epithelium volume fraction but separate optical properties. The reduced scattering coefficient was the least accurate predictor for both epithelium and stroma, suggesting this parameter has the least sensitivity distinguishing cellular versus connective glandular tissue. This is expected as reduced scattering is related simply to scatter density rather than a scatter size scale feature, like the scatter slope or phase function parameter, which is sensitive to changes in Rayleigh like scattering from collagen and Mie like scattering from cellular organelles. The root mean square error (rmse) and degree of freedom adjusted coefficient of determination ($r_{\mathrm{adj}}^{2}$) were calculated for each relationship and are shown in Figs. 2(a)–2(c). The mean rmse over all models was 0.16, whereas the mean $r_{\mathrm{adj}}^{2}$ was 0.69.

### 2.5 Statistical Methods

For the leave-one-out cross-fold validation (LOO-CV), performance metrics were calculated for all specimen lesion pixels and also specimen averaged values. The sensitivity, specificity, and accuracy were calculated for each of the three output classes for specimens and pixels, and CIs were calculated using the Clopper-Pearson method according to a binomial distribution using the MATLAB (v2016a) function *binofit*. A two-sided two-sample t-test was used to test statistical significance of differences in distributions.

## 3 Results

### 3.1 Histological Predictions from Label-Free Scatter Images

**Fig. 3:**
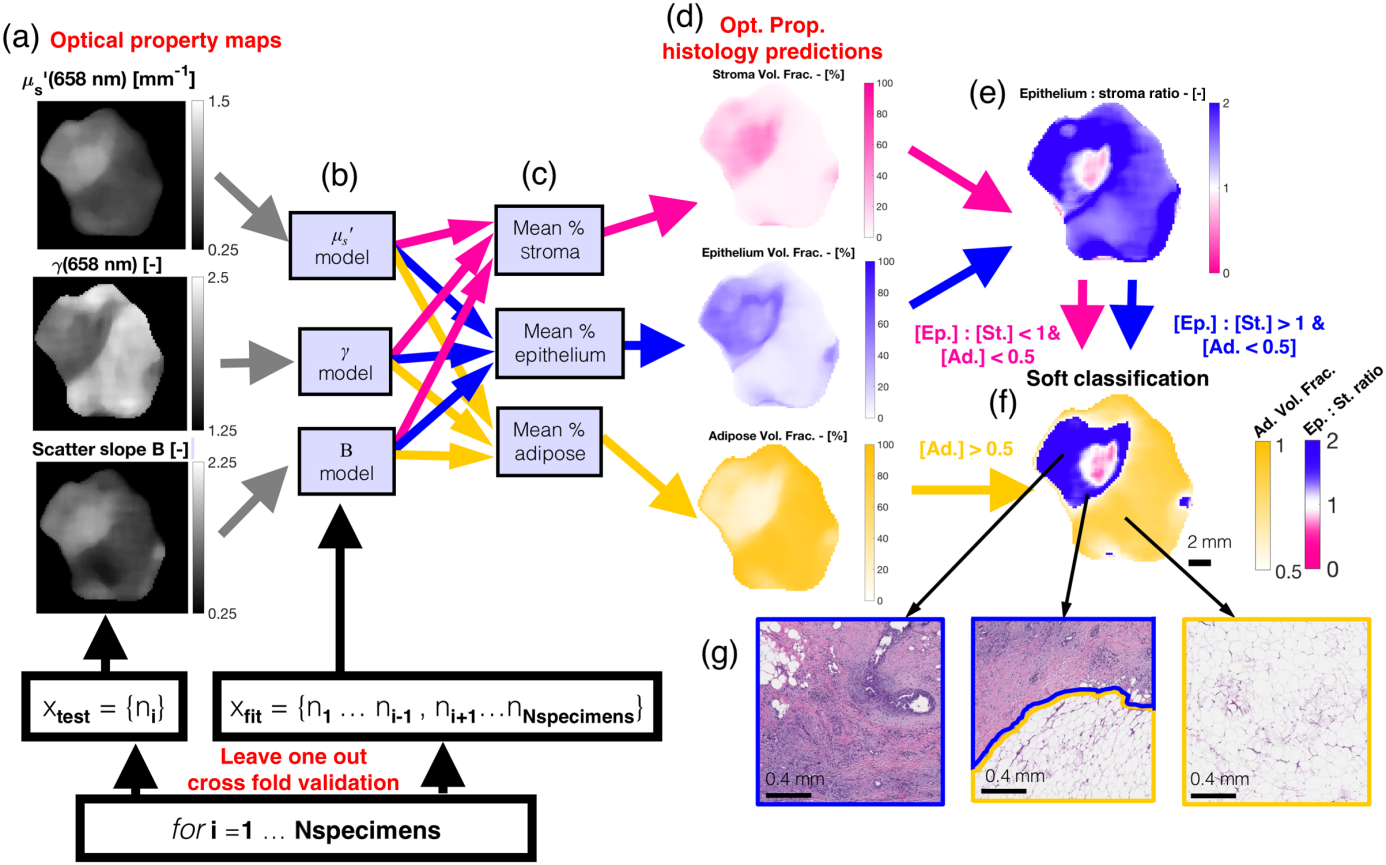
(a) Optical property maps of a test specimen. (b) Each optical property model is fitted with all specimens except the test specimen. This process is repeated over each specimen for the LOO-CV. (c) The histological volume fractions are predicted from the three optical property models and averaged together, resulting in the histology prediction maps in (d). A predicted epithelium to stroma ratio (Ep.: St. Ratio) is calculated in (e), which is simply the ratio of the epithelium and stroma predictions. In (f), a soft classification map is shown where each pixel is one of three colors for a classification of malignant, benign, or fat based on a threshold of 1 for the epithelium to stroma ratio and 0.5 for adipose volume fraction. The color saturation is varied based on how close the epithelium to stroma ratio and adipose volume fraction are close to their respective thresholds. In (g), H&E sections are shown for areas within the malignant lesion, on the border of the lesion, and for the background fat, confirming the optical property predictions.

Based on the fitted models described in Sec. 2.4, a predictive model of the epithelium, stroma, and adipose fractions as a function of the light scattering parameters was created and tested, in addition to a simple threshold-based tissue classification model as shown in Fig. 3. To test the accuracy of the predictive model, a LOO-CV scheme was used. For a given test specimen, such as Fig. 3(a), the models, Fig. 3(b), were fitted without the test data point, and this process was repeated over all specimens. The stoma, epithelium, and adipose fractions, shown in Fig. 3(d), were simply calculated as the mean of the predicted values from each of the three light scattering properties as shown in Fig. 3(c).

From these optical property predicted histological fractions, a simple threshold-based tissue classification scheme was implemented. Pixels with adipose volume fractions >50% were classified as fat. For pixels with adipose <50%, the ratio of epithelium to stroma (Ep.: St. Ratio), shown in Fig. 3(e), was used to classify remaining pixels as malignant or benign based on whether there was more epithelium than stroma or vice-versa. The epithelium to stroma ratio is displayed from 0 to 2, where white represents equal fractions of epithelium to stroma, pink represents no epithelium, and purple represents twice as much epithelium as stroma. The motivation for this manually selected classification scheme was to yield a simple biological interpretation of the histological predictions. Fat tissues were first classified from glandular components (both benign and malignant) by the adipose fraction being greater or less than the combined epithelium and stroma fraction. Within the glandular tissues, malignant and benign components were classified by the greater of the epithelium or stroma fractions, under the hypothesis that greater cellularity versus connective components was correlated to malignancy status. A “soft” classification map is shown in Fig. 3(f), where the color of each pixel is determined by the classification and the color saturation is determined by the continuous histological fractions. From this classification map, regions of the malignant lesion, the lesion bordering with fat, and a region of pure adipose are confirmed from the H&E section. Furthermore, a small false-negative region within the larger malignant lesion can be seen.

**Fig. 4:**
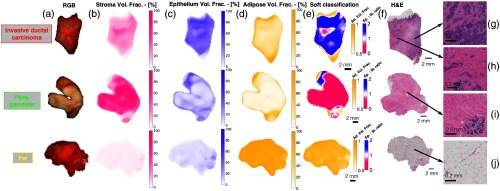
(a) Color photographs of representative invasive cancer, fibroglandular, and fat specimens. (b)–(d) Stroma, epithelium, and adipose volume fractions predicted from the optical property maps are shown, respectively, (e) with corresponding classification maps shown. (f) Whole specimen digitized H&E sections are shown, (g)–(j) while representative regions within each specimen are shown, which confirm the prediction of malignant, benign, and fat, respectively.

Case examples for a typical invasive ductal carcinoma (IDc), fibroglandular, and fat specimen are shown in Fig. 4. The color images, along with the optical property predicted stroma, epithelium, and adipose volume fraction maps, are shown in Figs. 4(a)–4(d), respectively, for each specimen. As expected, the IDc, fibroglandular, and fat specimens presented uniformly high epithelium, stroma, and adipose volume fractions, respectively. The resulting soft classification maps are shown in Fig. 4(e), which broadly corresponded to each specimens’ known pathology as confirmed by the whole specimen digitized H&E sections shown in Fig. 4(f). Representative regions within each section are shown in Figs. 4(g)–4(j), highlighting typical microscopic features associated with each pathology. There was a small false negative region within the IDc soft classification map, suggesting a stronger optical signal from extracellular stroma than cellular epithelium, as noted by the small pink region in the map indicating a predicted epithelium to stoma ration <1, and thus a misclassification of benign. Interestingly, the H&E section corresponding to this region, shown in Fig. 4(h), revealed an increased proportion of stroma, compared with the more cellular section, shown in Fig. 4(g), despite both regions coming from the same invasive ductal lesion. Thus, in this area of the lesion, despite a correct prediction of elevated stroma, the epithelium to stroma ratio at a threshold of 1 was an imperfect predictor of malignant tissue. Additionally, a slight artifact can be seen in the fibroglandular soft classification map between the transition fat to benign tissue at the specimen periphery. This false positive region is purple indicating a predicted epithelium to stoma ration >1, and thus a misclassification of malignant.

### 3.2 Correlation between Optical Property Predicted and H&E Segmented Metrics

**Fig. 5:**
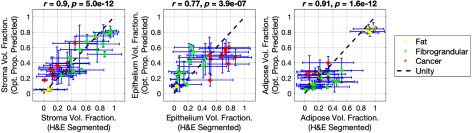
Plots of the H&E segmented histological volume fractions versus the optical property predicted volume fractions, calculated using a LOO-CV. Data points represent means of each specimen, while the H&E segmented error bars represent one standard deviation within each specimens and the optical property predicted error bars represent the standard deviation of the predicted values from each optical property.

The quantitative results of the LOO-CV analysis to test the accuracy predicting stroma, epithelium, and adipose volume fractions from optical property measurements are shown in Fig. 5. The optical property model and histological predictions are plotted as a function of the corresponding H&E segmented values, with data-points and error-bars representing the mean and standard deviation within each specimen, respectively. The resulting linear relationships provided evidence that the optical property predictions were strongly correlated to the values obtained from the digitized H&E slides, as quantified by the Pearson’s correlation coefficients (stroma r=0.90, $p<1\times 10^{-11}$, epithelium r=0.77, $p<1\times 10^{-6}$, adipose r=0.91, $p<1\times 10^{-11}$).

**Fig. 6:**
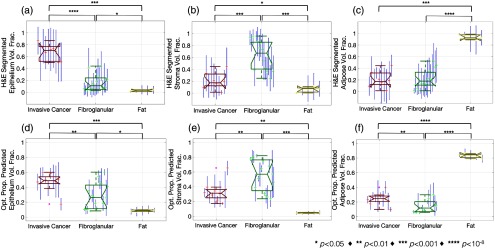
(a)–(c) Boxplots of H&E segmented epithelium, stroma, and adipose volume fractions are shown, respectively, (d)–(f) while corresponding boxplots of optical property predicted epithelium, stroma, and adipose volume fractions are shown, respectively. Data points represent means of each specimen, while the H&E segmented error bars represent one standard deviation within each specimen and the optical property predicted error bars represent the standard deviation of the predicted values from each optical property. For each pair of classes, a p-value range is shown calculated from two-sample student’s t-tests.

A complete report of the distributions of both optical property predicted and H&E segmented epithelium, stroma, and adipose volume fractions stratified by invasive cancer, fibroglandular, and fat pathologies is shown in Fig. 6. This confirmed that invasive cancer specimens presented a significantly higher mean epithelium volume fraction than fibroglandular as measured from digitized histology ($p<10^{-8}$) and the optical property model (p<0.01) shown in Figs. 6(a) and 6(d), respectively. Conversely, fibroglandular specimens presented a significantly higher mean stroma volume fraction than invasive cancer as measured by from digitized histology (p<0.001) and the optical property model (p<0.01) shown in Figs. 6(b) and 6(e), respectively.

### 3.3 Benign Versus Malignant Classification Performance

**Fig. 7:**
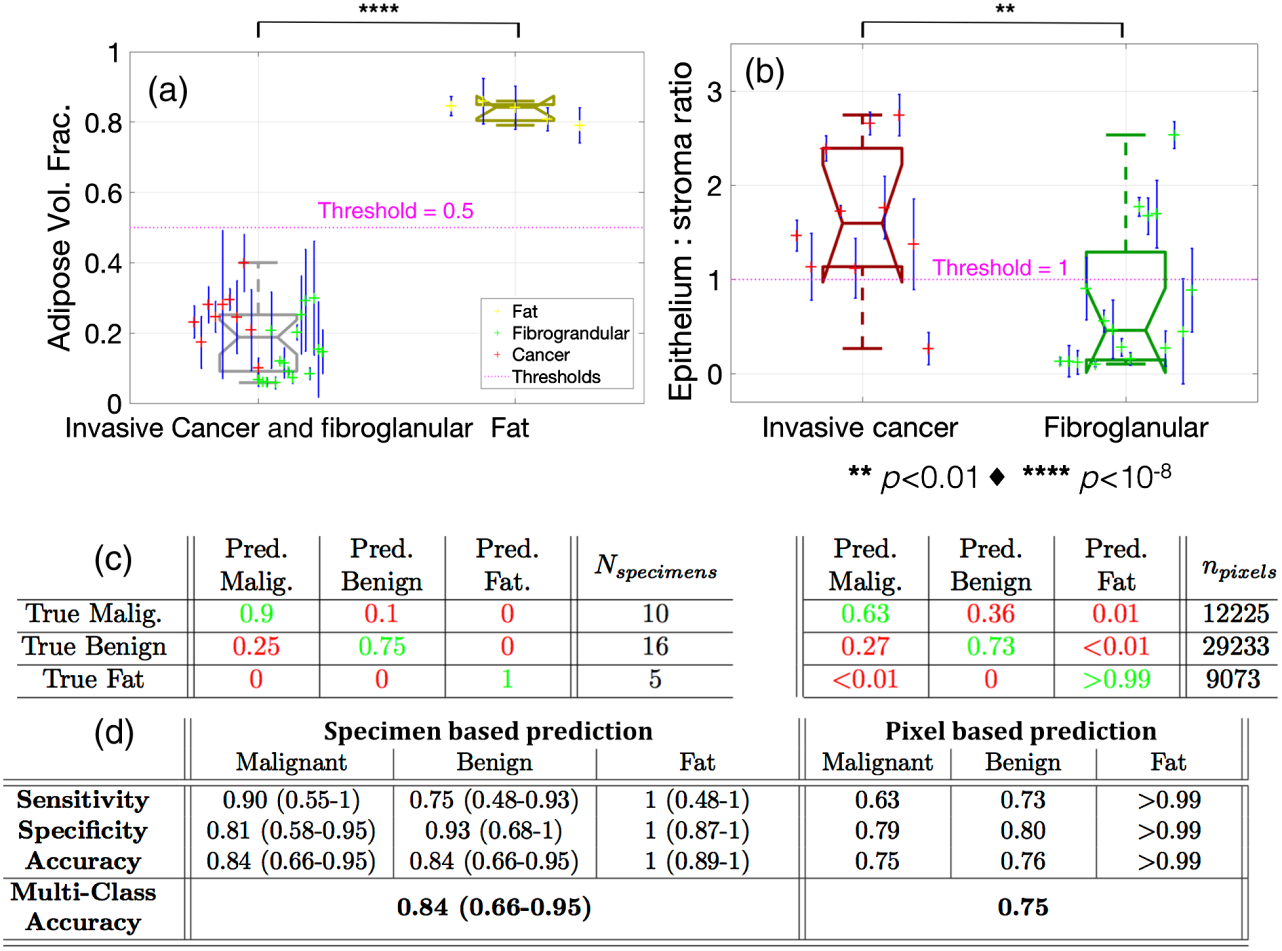
(a) Boxplot of the optical property predicted adipose volume fraction between glandular specimens (both malignant and benign) versus fat specimens. (b) Boxplot of the optical property predicted epithelium to stroma ratio between malignant and benign specimens. Thresholds used for classification are shown in magenta and p-values of a two-sample t-test are shown in black. (c) Confusion matrices that display the fraction of correctly classified data points in green and those incorrectly classified in red. (d) Performance tables listing classification performance metrics. In (c) and (d), the left table represents specimen averaged classification, whereas the right table represents classification over all individual pixels. Values in parenthesis represent Clopper–Pearson 95% CIs, while no parenthesis represents 95% CIs are within ±1%. All prediction and classification data were obtained with LOO-CV.

A summary of the quantitative performance analysis of the tissue classification algorithm is shown in Fig. 7. The optical property predicted adipose volume fraction boxplots in Fig. 7(a) showed a clear separation between the fat specimens and the aggregated invasive cancer and fibroglandular specimens at a threshold of 0.5 ($p<1\times 10^{-14}$). But more importantly, the optical property predicted epithelium to stroma ratio boxplots in Fig. 7(b) showed a clear separation between the invasive cancer and fibroglandular specimens at a threshold of 1 (p=0.007). For all boxplots, the means of each specimen are plotted with error-bars representing one standard deviation within each specimen, characterizing intraspecimen variation.

Confusion matrices tabulating the fraction of true versus predicted classifications of all specimens (left) and pixels (right) are shown in Fig. 7(c). The values along the diagonals of each matrix denote the fraction of correctly classified specimens or pixels for a given class, while off-diagonal values denote those incorrectly classified. The sensitivity, specificity, and accuracy were quantified from these data and are tabulated in Fig. 7(d). While the fraction of correctly classified malignant specimens was 0.9 when specimen averaging is applied, the fraction of correctly classified malignant pixels decreases to 0.63. Furthermore, when the specimens mean data are classified versus all pixels within each specimen lesion, the malignant sensitivity decreases from 0.9 to 0.63 and the benign specificity decreases from 0.93 to 0.8. The apparent heterogeneity in cellular versus stromal densities in malignant lesions suggests that lesion-based averaging may increase robustness. Nevertheless, the overall accuracy of correctly classifying a given pixel was 0.75, whereas the overall accuracy of classify a given specimen was 0.84.

### 3.4 Spatial Quantification of Histological Metrics and Tissue Classification

**Fig. 8:**
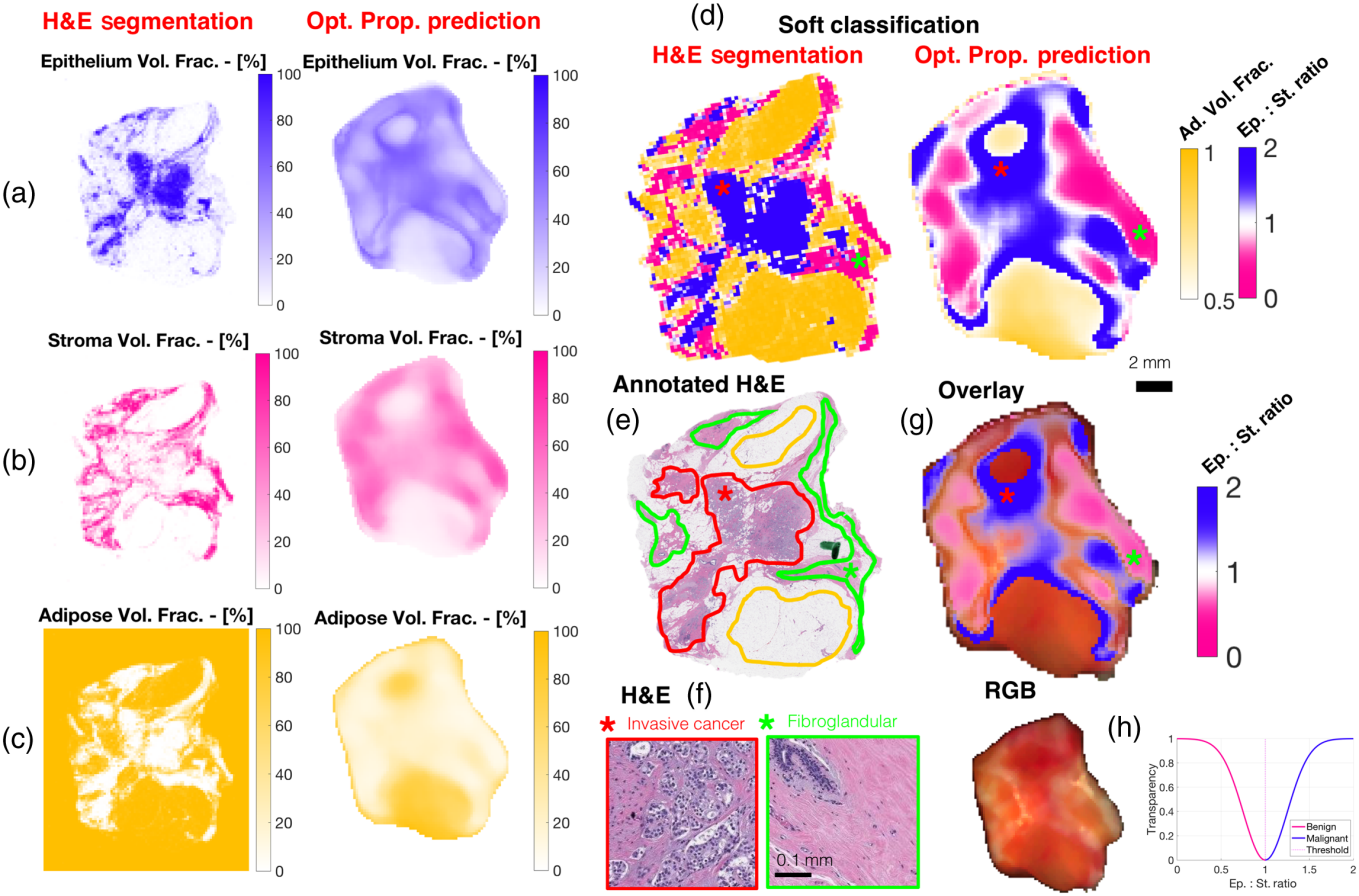
Side-by-side comparisons of H&E segmented and optical property predicted epithelium, stroma, and adipose volume fraction maps in (a), (b), and (c), respectively, for a specimen with malignant and benign regions. In (d), H&E segmented and optical property predicted classification maps are shown, and a corresponding H&E section of the entire specimen is shown in (e) with outlined malignant, benign, and fat regions. (f) Zoomed in H&E sections of invasive ductal carcinoma and benign connective tissue regions are shown. (g) An overlay of the epithelium to stroma ratio onto the photograph of the specimen is shown, (h) with a graph of the overlay transparency and original photograph of the specimen.

Side-by-side comparisons of the model predicted metrics and digitized H&E data are shown for a heterogeneous specimen in Fig. 8. Spatially resolved maps of H&E segmented and optical property predicted epithelium, stroma, and adipose volume fractions are shown side by side in Figs. 8(a)–8(c), respectively. Soft classification maps, shown in Fig. 8(d), were calculated from both the H&E segmented and optical property predicted histological metrics. For both the soft classification and histological metrics, similar spatial features were seen in both the H&E segmented and optical property predicated maps, despite the notable difference in spatial resolution and expected minor co-registration differences arising from specimen dehydration, fixation, and sectioning. The whole digitized H&E slide is shown in Fig. 8(e), with malignant, benign, and fatty regions outlined. The presence of these outlined lesions can be seen in both of the soft classification maps, albeit with some spatial noise. Representative sections of the malignant invasive lesion and benign fibroglandular region are shown in Fig. 8(f), the locations of which are marked by red and green asterisks, respectively in both the soft classification maps, Fig. 8(d), and the annotated H&E slide, Fig. 8(e). A simple overlay of the optical property predicted soft classification map onto the white light image is shown in Fig. 8(g). The two image sets are inherently coregistered as they were acquired with the same imaging system. The original specimen color image along with the graph of the overlay transparency is shown in Fig. 8(h). When epithelium and stroma have a similar predicted strength, and thus the classification is less certain, the overlay becomes transparent. The adipose volume fraction was not overlaid as fat is distinguishable by inspection.

## 4 Discussion and Conclusions

Label-free light scattering measurements have shown sensitivity to morphological changes between malignant and benign tissues through a multitude of studies across a myriad of optical sensing devices over the last 20 years.^12^^–^^16^^,^^27^^–^^36^ However, in all of these studies, light scattering is quantified either empirically or with physical radiative transport terms, both of which lack biological or clinical meaning. Therefore, the aim of this study was to determine if quantitative light scattering measurements could be related to and predictive of clinically relevant histological metrics, specifically the volume fraction of stroma, epithelium, and adipose. This hypothesis was systematically tested by investigating the relationship between optical scattering properties of freshly resected breast tissue determined from SFDI to histology metrics segmented from coregistered and digitized H&E sections of whole specimens. Logistic and Gaussian responses were observed between the optical scattering properties and volume fractions of stroma, epithelium, and adipose. From these observations, a simple model demonstrated that in fact common optical properties could explicitly predict and spatially map volume fraction of stroma, epithelium, and adipose, which in turn provided diagnostic accuracy in predicting malignant from benign lesions.

Previous studies utilizing spatially constrained spectroscopic reflectance measurements have revealed the sensitivity of light scattering to breast tissue morphologies. Laughney et al.^14^ have measured 47 BCS specimens with diffuse SFDI, which yielded chromophore concentration and scatter parameter maps, and demonstrated that the scatter slope and amplitude were the most discriminating features in their classification algorithm. Furthermore, Wilke et al.^37^ demonstrated in a cohort of 55 BCS specimen margins measured with diffuse reflectance spectroscopy, that the ratio of the spectrally averaged scattering to beta-carotene, a chromophore giving adipose its yellow color, was the most diagnostically discriminate parameter. Interestingly, hemodynamic parameters were less sensitive, with the possible explanation that superficial blood on the specimen margin may originate from the surgery and be nonspecific to the tumor. Additionally, simple RGB imaging has shown sensitivity to murine tissue morphology, if specular reflections are removed with cross polarizers,^38^ but a full analysis is yet to be conducted in human breast tissue. Similarly, the presence of surface blood could likewise hinder RGB sensitivity during surgery.

The physical origins of light scattering arise from spatial fluctuations in the refractive index over size scales both smaller and larger than the wavelength of light, each of which uniquely contribute to the spectral and angular intensities of light scattering.^22^^,^^23^^,^^39^^–^^41^ Although these fluctuations cannot be resolved with a conventional microscope, the staining of hematoxylin and eosin allows for an estimate of the relative proportions of stroma, epithelium, and adipose, each of which have very unique ultrastructural size-scale features. This has been demonstrated in previous studies where fibroglandular tissues have presented both increased scattering intensity and increased collagen content when compared with more cellular malignant lesions as measured in human breast tissue with phase contrast microscopy^33^ and dark-field microscopy,^29^ as well as in human ovarian tissue measured with optical coherence tomography.^32^ Furthermore, weak positive correlations have been reported between spectroscopic scattering and the fraction of stroma or collagen measured by spatial frequency domain imaging^14^ and diffuse reflectance spectroscopy.^34^ However in this study, light scattering was further decoupled into spectroscopic and angular components, which was achieved by increased signal localization with high spatial frequency illumination, allowing for more sensitive measurements.^15^^,^^19^

Although strong correlations between optical property predicted and H&E segmented histology metrics were found and shown to be diagnostically relevant, there are a few notable limitations with this technique. First the spatial correlations between the optical images and the H&E slide are not exact, as the tissue shrinks during the fixation process. Although lesion-based averaging was used to overcome this common limitation, a further issue is depth coregistration, as the H&E section represents a superficial slice of the tissue only a few microns in thickness, while the optical measurements have a depth sensitivity of a few hundred microns. The inexactness of the depth coregistrations was likely the dominant source variability in the model. Second, the endpoints of stroma, epithelium, and adipose volume fractions are imperfect for both the description for origins of scattering signals and the metrics to classify benign from malignant lesions. This imperfection was highlighted in Figs. 3(f) and 4(e), as regions of stromal proliferations within malignant lesions lead to erroneous false negative classifications. Additionally, the color-based stain segmentation to estimate the relative fractions of epithelium, stroma, and fat, assumes that the breast specimens are comprised of these components, when in fact other pathologies exist. However, this simplification enables an estimation of scattering contributions from collagen, cellular features, and adipose, which have very distinct scattering features.

As mentioned previously, there are many ultrastructural features not quantified through histology, such as chromatin packing, mitochondria density, and collagen reformation, which could greatly affect both the light scattering properties and pathological diagnosis. One such example is that benign associated stroma in human breast tissue was shown to be more strongly scattering than tumor associated stroma.^33^ However, further studies could stratify benign and malignant regions into levels of organization or grade as a proxy for neoplastic architectural changes. Furthermore, classification thresholds were manually chosen for biologic simplicity to be the largest fraction of adipose or glandular components to segment fat, and the greatest fraction of epithelium or stroma within glandular components to segment malignant from benign tissue. But in the future, more complex classification schemes could be automated and may result in improved performance. A practical limitation to clinical translation is the processing time to spatially map optical properties, which is currently ∼1  s/pixel on a laptop. However, a recently published method has demonstrated mapping times of ∼10  ms/pixel using dense lookup-tables, which could be implemented in the future.^42^ Additionally, SFDI does require a projector to create the structured illumination, but SFDI studies have been performed with an off-the-shelf, inexpensive commercial projector, which could lower the cost and complexity of the imaging system.^38^^,^^43^ Despite these limitations, this study was able to show that these scatter parameters acquired from fresh, unprocessed, and unlabeled tissue could be used to quantified morphological parameters, which can currently only be obtained through timely histopathology processing.

In conclusion, it was shown that label-free light scattering measurements of freshly resected human breast tissue acquired with SFDI were explicitly related to and predictive of clinically relevant histology metrics, as quantified from digitized, whole specimen H&E slides. Three optical properties related the scatter density, spectroscopic scattering intensity, and directional scattering intensity were found to have a logistic relationship to the stroma and adipose volume fraction and a Gaussian relationship to the epithelium volume fractions. From these relationships, a predictive model was created and validated with LOO-CV, which demonstrated that the optical property predicted epithelium to stroma ratio was diagnostically relevant in distinguishing malignant from benign glandular tissue. With future development, this technology may aid in the surgical triaging of large, freshly resected BCS specimens.
